# Retrospective analysis of postoperative complications following surgical treatment of ileal impaction in horses managed with manual decompression compared to jejunal enterotomy

**DOI:** 10.3389/fvets.2023.1156678

**Published:** 2023-04-27

**Authors:** Jennifer Ruff, Sandra Zetterstrom, Lindsey Boone, Erik Hofmeister, Caitlin Smith, Kira Epstein, Anthony Blikslager, Callie Fogle, Megan Burke

**Affiliations:** ^1^Department of Clinical Sciences, North Carolina State University, Raleigh, NC, United States; ^2^Department of Clinical Sciences, Auburn University, Auburn, AL, United States; ^3^Department of Clinical Sciences, New Bolton Center, University of Pennsylvania, Philadelphia, PA, United States; ^4^Department of Clinical Sciences, University of Georgia, Athens, GA, United States

**Keywords:** colic, ileum, impaction, enterotomy, manual decompression

## Abstract

**Objective:**

The objective of this study was to compare the occurrence of post-operative complications and survival to discharge in horses with ileal impactions resolved by manual decompression compared with jejunal enterotomy.

**Animals:**

A total of 121 client-owned horses undergoing surgical correction of an ileal impaction at three teaching hospitals.

**Materials and methods:**

Data from the medical records of horses undergoing surgical correction of an ileal impaction was retrospectively collected. Post-operative complications, survival to discharge, or post-operative reflux present were evaluated as dependent variables and pre-operative PCV, surgery duration, pre-operative reflux, and type of surgery were evaluated as independent variables. Type of surgery was divided into manual decompression (*n* = 88) and jejunal enterotomy (*n* = 33).

**Results:**

There were no significant differences in development of minor complications, development of major complications, presence of post-operative reflux, amount of post-operative reflux, and survival to discharge between horses that were treated with manual decompression and those treated with distal jejunal enterotomy. Pre-operative PCV and surgery duration were significant predictors of survival to discharge.

**Conclusions and clinical relevance:**

This study showed that there are no significant differences in post-operative complications and survival to discharge in horses undergoing distal jejunal enterotomy versus manual decompression for correction of ileal impaction. Pre-operative PCV and duration of surgery were found to be the only predictive factors of survival to discharge. Based on these findings, distal jejunal enterotomy should be considered earlier in horses with moderate to severe ileal impactions identified at surgery.

## Introduction

**Figure 1:**
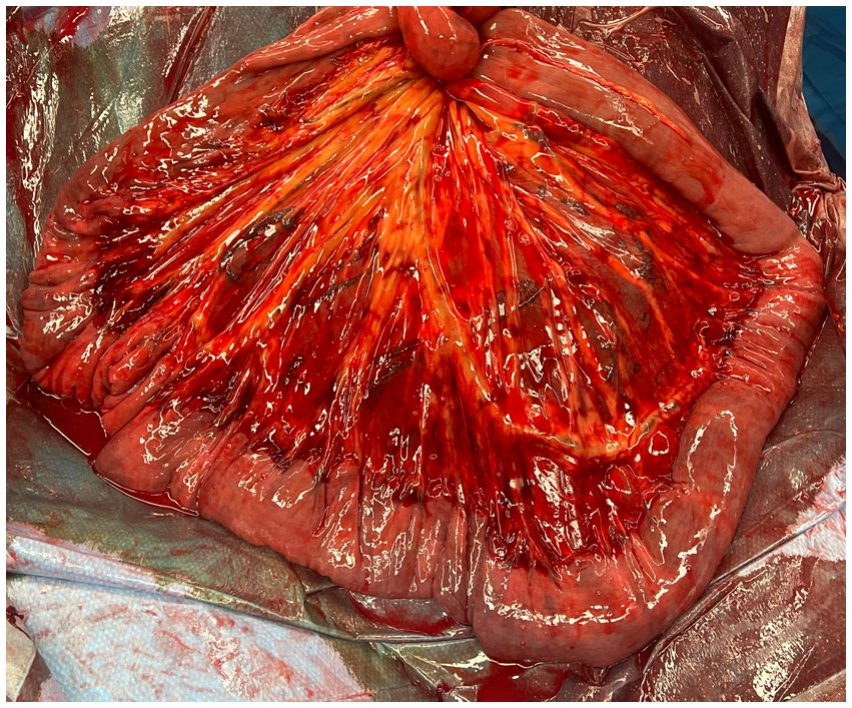
Serosal and mesenteric inflammation of the ileum and distal jejunum after manual decompression to resolve an ileal impaction.

Ileal impaction is the most common cause of non-strangulating obstruction of the equine small intestine, accounting for 2.2–23% of all small intestinal colic cases (1). Ileal impactions are often successfully resolved with medical treatment. However, horses with persistent abdominal pain, peritoneal fluid abnormalities, and gastric reflux may require surgical intervention (2). A commonly described surgical method is manual decompression by extraluminal massage of the impaction and passage of ingesta into the cecum. Intraluminal infusion of saline, or dioctyl sodium sulfosuccinate (DSS), as well as hydration of the impaction with fluid from the proximal bowel, have all been described to aid surgical resolution (3). Despite widespread use of manual decompression, the amount of tissue handling necessary and potential for increased surgery time may predispose horses to complications such as adhesion formation and post-operative ileus due to increased inflammation in the intestinal wall (4–9) (Figure 1).

Enterotomy of the small intestine is rarely performed, due to concern for contamination of the surgical field, the potential for prolonging the return of peristalsis, adhesion formation, and a risk of stricture at the enterotomy site (10). Performing a jejunal enterotomy may decrease tissue trauma as a previous study showed that performing a jejunal enterotomy created a less severe inflammatory reaction than other types of small intestinal manipulation (11). This may be especially beneficial in more severe cases requiring excessive manipulation, or in young horses more prone to adhesions. A recent case report describes a good outcome following jejunal enterotomy for resolution of an ileal impaction in a 4 month old Arabian filly (12). Previous retrospective studies have included a small number of cases where distal jejunal enterotomies were performed to aid in reduction of ileal impactions (2, 10, 13–16). However, no studies have specifically investigated the use of jejunal enterotomy to treat ileal impaction. Furthermore, no study has compared the rate of complications or survival outcomes for horses undergoing surgical correction of ileal impaction using manual reduction versus jejunal enterotomy.

It is our clinical impression that performing a jejunal enterotomy decreases surgical time and tissue handling, which may decrease postoperative complications and improve long term outcomes. The objective of the present study was to compare the occurrence of post-operative complications and survival to discharge in horses with ileal impactions resolved by manual decompression with those resolved using jejunal enterotomy. Our first hypothesis was that horses treated with distal jejunal enterotomy would have fewer postoperative complications than horses treated with manual decompression. We further hypothesized that horses undergoing distal jejunal enterotomy would have increased survival to discharge than horses treated with manual decompression.

## Materials and methods

### Inclusion criteria

Horses that had surgical correction for an ileal impaction at three equine teaching hospitals between January 1, 2008 and May 1, 2021 were included in the study. Horses were excluded if the ileal impaction was not the primary lesion identified at surgery or if the horse was euthanized intraoperatively.

### Pre-operative information

Pre-operative information obtained from the medical record included admission date, age, sex, breed, surgery date, duration of colic episode, admission physical exam (heart rate, respiratory rate, temperature, mucous membrane color, capillary refill time, borborygmi), admission bloodwork (packed cell volume, total protein, lactate, white blood cell count), abdominal palpation per rectum findings, abdominocentesis (gross appearance, lactate, protein, white blood cell count), abdominal ultrasound findings, and presence/absence of reflux (defined as >2 L obtained at any point before surgical intervention).

### Intra-operative information

Intra-operative information gathered from the medical record included anesthesia and surgery time and manual decompression vs. jejunal enterotomy.

### Post-operative information

Post-operative information obtained included presence/absence of post-operative reflux (POR) (defined as >2 L of reflux at any intubation following surgery), post-operative colic, repeat laparotomy, systemic inflammatory response (SIRS), defined as having 3 or more of the following: heart rate > 70 beats per minute, packed cell volume > 45%, temperature > 101.5*F within 48 h post-operatively, white blood cell count of >12,500 cells/μL or < 4,500 cells/μL, and a respiratory rate of >30 breaths per minute, incisional infection, and post-operative euthanasia.

Complications other than post-operative reflux were categorized as either minor or major. Minor complications included postoperative colic that resolved without surgery and SIRS. Major complications included the need for a second laparotomy and development of incisional infection.

### Statistics

Logistic regression models with backwards conditional elimination using minor complications, major complications, survival to discharge, or post-operative reflux present as dependent variables and pre-operative PCV, surgery duration, pre-operative reflux, and type of surgery as independent variables were built (four different models). A linear regression model with backwards elimination using post-op reflux amount as the dependent variable and pre-operative PCV, surgery duration, pre-operative reflux, and type of surgery as independent variables was built. Independent variables were chosen by the authors as being those believed to be most relevant to the patient outcome. A ROC curve for pre-operative PCV and surgery duration was built to distinguish patients who survived vs. those who did not. The Youden’s Index was calculated to determine the break point value which best predicted patient survival. Cases were then divided into being above or below this break point (i.e., “long” surgery duration or “not long” surgery duration) and a new logistic regression model was built with survival as the dependent variable and pre-operative PCV (“high” or “not high”), surgery duration (“long” or “not long”), pre-operative reflux, and type of surgery as independent variables. Significance was set at alpha = 0.05.

## Results

One hundred and thirty horses underwent surgical correction of an ileal impaction at one of the three equine referral centers between January 1, 2008 and May 1, 2021. Ten horses were euthanized intraoperatively and therefore excluded. One hundred and five cases had all of the necessary data for multivariate analysis. Breeds included 35 Quarter Horses, 17 Thoroughbreds, 9 Warmbloods, 7 Arabians, 6 Paints, 4 Irish Sport Horses, 4 Morgans, 4 Saddlebreds, 3 Friesians, 3 Gypsy Vanners, 3 Hanoverians, 3 Oldenburgs, 3 Percherons, 3 Paso Finos, 2 Holsteiners, 2 Missouri Fox Trotters, 2 Mixed Breed horses, 2 Ponies, 2 Welsh Ponies, 1 American Saddle Horse, 1 Andalusian, 1 Belgian, 1 Dutch Warmblood, 1 Mecklenberg, 1 Standardbred, and 1 unknown breed. Median age was 10 years (range, 0.42–28 years).

No preoperative variables were significantly different when compared between horses treated with manual decompression vs. jejunal enterotomy (Table 1). No preoperative variables were associated with the development of minor or major complications, or with the presence or volume of post-operative reflux. There were no significant differences in development of minor complications, development of major complications, presence of post-operative reflux, amount of post-operative reflux, and survival to discharge between horses that were treated with manual decompression and those treated with distal jejunal enterotomy (Table 2).

**Table 1:** Results of univariable analysis of pre-operative variables between decompression and enterotomy groups.

	Manual decompression (*n* = 88)	Jejunal enterotomy (*n* = 33)	*p*-value
Lactate (mmol/L)	2.7 ± 2.2 (2.2–3.2)	3.4 ± 3.3 (2.1–4.7)	0.54
Heart rate (bpm)	58 ± 20 (53–62)	59 ± 22 (51–67)	0.93
Peritoneal fluid lactate (mmol/L)	4.0 ± 2.8 (3.2–4.9)	4.6 ± 3.9 (2.3–6.9)	0.73
Peritoneal fluid protein (mmol/L)	2.5 ± 1.1 (2.2–2.9)	2.9 ± 1.3 (2.1–3.6)	0.34
Colic duration (hours)	2.4 ± 0.9 (2.2–2.6)	2.5 ± 0.9 (2.2–2.9)	0.49
Respiratory rate (brpm)	23 ± 8.7 (21–25)	29 ± 14 (21–31)	0.82
Temperature (°F)	99.5 ± 1.2 (99.2–99.7)	99.3 ± 1.8 (98.7–100.0)	0.77
Packed cell volume (%)	42 ± 8 (40–44)	42 ± 7 (39–45)	0.91
Total protein (g/dL)	7.5 ± 1.1 (7.3–7.7)	7.3 ± 0.9 (7.0–7.6)	0.4
White blood cell count (×10^3^/μL)	9.3 ± 3.4 (8.6–10)	9.6 ± 3.5 (8.2–11)	0.7
Peritoneal fluid white blood cell count (×10^3^/μL)	1,142 ± 3,406 (193–2090)	746 ± 2,130 (−389–1880)	0.66
Reflux (L)	6.9 ± 8.6 (4.5–9.2)	4.5 ± 7.9 (1.2–7.7)	0.22
CRT (seconds)			0.1
<2 s	40 (47%)	10 (29%)	
>2 s	46 (53%)	24 (71%)	
Borborygmi			0.49
Normal	5 (6%)	4 (12%)	
Decreased	55 (65%)	19 (58%)	
Absent	25 (29%)	10 (30%)	
Distended small intestine on rectal palpation			1
Yes	76 (90%)	27 (93%)	
No	8 (10%)	2 (7%)	
Ileal impaction palpable			0.72
Yes	7 (9%)	3 (11%)	
No	73 (91%)	25 (89%)	
Grossly turbid peritoneal fluid			0.3
Yes	18 (23%)	4 (13%)	
No	61 (77%)	27 (87%)	
Grossly serosanguinous peritoneal fluid			0.76
Yes	12 (15%)	3 (10%)	
No	68 (85%)	27 (90%)	
Distended small intestine on ultrasound examination			0.3
Yes	82 (98%)	30 (94%)	
No	2 (2%)	2 (6%)	
Reflux			0.3
Yes	32 (35%)	15 (45%)	
No	59 (65%)	18 (55%)	

Values are expressed as mean (± standard deviation) for continuous variables.

**Table 2:** Dependent and independent variables evaluated for each surgical treatment group.

	Manual decompression (*n* = 88)	Jejunal enterotomy (*n* = 33)	*P*-value
Age (years)	9.46 (5.99)	12.98 (6.72)	0.62
Pre-operative PCV (%)	42.2 (8.45)	42.2 (7.10)	0.002
Pre-operative reflux	30/88 (34%)	14/33 (42.2%)	0.29
Surgery duration (minutes)	132.5 (44.11)	146.9 (39.72)	0.025
Post-operative reflux	46/88 (52.3%)	18/33 (54.5%)	0.30
Volume of post-operative reflux (L)	24.2 (57.44)	33.4 (56.76)	0.45
Minor complications	51/88 (58%)	18/33 (54.5%)	0.68
Major complications	19/88 (21.6%)	3/33 (9.1%)	0.12
Survival to discharge	69/88 (78.4%)	25/33 (75.8%)	0.45

Values are mean (± standard deviation). *p* < 0.05 indicates a significant effect on survival to discharge.

Pre-operative PCV (*p* = 0.002) and surgery duration (*p* = 0.025) were significant predictors of survival to discharge. The ROC curve indicated a cut off to discriminate surviving and non-surviving horses for PCV at >51% and for surgery duration at >118.5 min. Horses with a high PCV were 13.7 times less likely (95% CI: 3.1–60.9) to survive (*p* = 0.001) and horses with a long surgery duration were 6.2 times less likely (95% CI: 1.5–25.5) to survive (*p* = 0.012).

## Discussion

This study compared survival to discharge, development of postoperative reflux, and the number and severity of postoperative complications in horses undergoing surgical correction of an ileal impaction with manual decompression versus jejunal enterotomy. There was no difference between groups in the development of reflux, or in the total volume of reflux produced. Similarly, there was no difference in the development of major or minor complications between groups.

Retrospective studies of horses with ileal impaction from the 1980s (10, 14), reported decreased survival for horses undergoing small intestinal enterotomy, compared to horses where no enterotomy was performed. However, a recent case report (12) showed good short-and long-term outcomes in a filly with an ileal impaction treated by jejunal enterotomy. In light of advances in veterinary medicine in the last 30 years, our aim was to reassess the safety and efficacy of jejunal enterotomy in a group of horses undergoing surgical correction of ileal impaction. Our results showed that for this group of horses, survival to discharge was not better for manual decompression compared with jejunal enterotomy.

Similar to previous studies (17–21), increased pre-operative PCV was found to be a negative predictor of survival to discharge. In our population, every 1% increase in PCV decreased the likelihood of survival to discharge by ~12%. Increased pre-operative PCV is often correlated with more severe hemodynamic compromise, due to either the duration of colic, or the severity of the lesion (21). Both of these factors make horses poorer anesthetic candidates and increased pre-operative PCV likely reflects the degree of systemic inflammatory response that may develop post-operatively (22). These horses are more likely to develop post-operative complications, increasing their morbidity and mortality rates.

Surgery time was also identified as a significant predictor of survival to discharge. Horses with surgery longer than 118.5 min were 6.2 times less likely to survive to discharge than horses with surgery time less than 118.5 min. A previous study (23) found that duration of surgery is a variable with considerable prognostic value on long-term survival. Some causes of longer surgery time (e.g., amount of affected bowel, accessibility of affected segments) are unavoidable. Other factors, such as rapid identification of the lesion and surgical decision making, can shorten surgery, indirectly improving survival. In our clinical experience, performing a jejunal enterotomy in horses with long or very firm impactions results in faster resolution of the impaction compared to manual decompression. In this study although there was no significant difference in surgical time between manual decompression and jejunal enterotomy, mean surgical time for the enterotomy group was longer than for manual decompression. It is likely, especially for the earlier cases in this retrospective, that many surgeons attempted manual decompression prior to performing an enterotomy and only transitioned to jejunal enterotomy once manual decompression failed. It is our clinical impression that resolution of the impaction occurs rapidly once enterotomy is performed, whether it is done immediately or after a period of manual decompression. As this study shows no negative effects of enterotomy on short term survival, we would suggest that transitioning to an enterotomy sooner in more severe cases may shorten surgery times or result in differences in outcome for horses undergoing manual decompression vs. enterotomy. However, a prospective study would be required to confirm this assumption.

Manual decompression increases intestinal inflammation due to increased tissue handling, which may lead to development of post-operative ileus. A previous study has evaluated different types of intestinal manipulation during surgery, including manual decompression and intestinal enterotomy. The results of that study demonstrated that emptying the small intestine via manual decompression causes more severe neutrophilic infiltration of the jejunal serosa and circular muscle than emptying via jejunal enterotomy in horses without gastrointestinal disease (11). Furthermore, although enterotomy alone did lead to an inflammatory response, it was weaker than all other intestinal manipulations performed (11). This supports performing an enterotomy sooner in cases of moderate to severe ileal impaction because reducing the amount of inflammation caused by surgical manipulation may decrease the risk of post-operative ileus, and result in better outcomes. The information gained from those studies suggests that a distal jejunal enterotomy should be considered as the first line of treatment in horses with moderate to severe ileal impactions.

Our hypothesis that distal jejunal enterotomy would decrease post-operative complications and result in greater survival to discharge compared to manual decompression was not supported. However, our results showed that jejunal enterotomy resulted in similar survival to discharge compared to manual decompression. Therefore, jejunal enterotomy is a valid alternative to manual decompression in horses requiring surgical resolution of ileal impactions.

One limitation of this study is the number of cases, particularly in the enterotomy group which may have resulted in Type 1 error. Analysis of a larger population of horses may have resulted in statistically significant differences in the post-operative complications and survival to discharge between the two groups. However, distal jejunal enterotomies have not been performed commonly until more recently, limiting the number of cases available. The retrospective nature of this study also limits data analysis to what is available in the medical record. Information on the length and severity of the ileal impactions was not routinely recorded, and would be useful in future studies to help determine if enterotomies are truly beneficial in more severe impactions. Prospective clinical studies with horses randomly assigned to receive manual decompression or jejunal enterotomy are also warranted to evaluate the effect of treatment on the development of postoperative complications and survival outcomes. These results also only consider the short-term outcome in these cases. Long-term follow up would allow for better evaluation of complications such as adhesion formation and long-term survival rates.

In conclusion, this study showed that there are no significant differences in post-operative complications and survival to discharge in horses undergoing distal jejunal enterotomy versus manual decompression for correction of ileal impaction. Pre-operative PCV and duration of surgery were found to be the only predictive factors of survival to discharge. Based on these findings, distal jejunal enterotomy should be considered earlier in horses with moderate to severe ileal impactions identified at surgery.

## Data availability statement

The raw data supporting the conclusions of this article will be made available by the authors, without undue reservation.

## Ethics statement

Ethical review and approval was not required for the animal study because this is a retrospective study. Written informed consent for participation was not obtained from the owners because this is a retrospective study.

## Author contributions

LB contributed to study design, data collection from residing institution, data review, manuscript preparation, and manuscript review. SZ contributed to study design, data collection from all institutions, and manuscript review. EH contributed to data analysis, manuscript preparation, and manuscript review. KE and CS performed data collection from residing institution and manuscript review. JR contributed to study design, data collection from all institutions, manuscript preparation and manuscipt review. MB contributed to study design, manuscript preparation, and manuscript review. All authors contributed to the article and approved the submitted version.

## Conflict of interest

The authors declare that the research was conducted in the absence of any commercial or financial relationships that could be construed as a potential conflict of interest.

## Publisher’s note

All claims expressed in this article are solely those of the authors and do not necessarily represent those of their affiliated organizations, or those of the publisher, the editors and the reviewers. Any product that may be evaluated in this article, or claim that may be made by its manufacturer, is not guaranteed or endorsed by the publisher.

## References

[1] Freeman DE. Jejunum and Ileum In: Auer JA Stick JA Kümmerle JM Prange T, editors. Equine surgery. 5th ed. St. Louis, MO: Elsevier (2019). 536–74.

[2] Fleming K Mueller POE. Ileal impaction in 245 horses: 1995-2007. Can Vet J. (2011) 52:759–63. doi: 10.1093/ajhp/52.7.759 22210940 PMC3119239

[3] Hanson RR Wright JC Schumacher J Baird AN Humburg J Pugh DG. Surgical reduction of Ileal impactions in the horse: 28 cases. Vet Surg. (1998) 27:555–60. doi: 10.1111/j.1532-950X.1998.tb00531.x, PMID: 9845219

[4] Mair TS Smith LJ. Survival and complication rates in 300 horses undergoing surgical treatment of colic. Part 3: long-term complications and survival. Equine Vet J. (2010) 37:310–4. doi: 10.2746/0425164054529445, PMID: 16028618

[5] Fogle CA Gerard MP Elce YA Little D Morton AJ Correa MT. Analysis of sodium carboxymethylcellulose administration and related factors associated with postoperative colic and survival in horses with small intestinal disease. Vet Surg. (2008) 37:558–63. doi: 10.1111/j.1532-950X.2008.00420.x, PMID: 19134106

[6] Roussel AJ Cohen ND Hooper RN Rakestraw PC. Risk factors associated with development of postoperative ileus in horses. J Am Vet Med Assoc. (2001) 219:72–8. doi: 10.2460/javma.2001.219.72, PMID: 11439775

[7] Cohen ND Lester GD Sanchez C Merritt AM Roussel AJ. Evaluation of risk factors associated with development of postoperative ileus in horses. J Am Vet Med Assoc. (2004) 225:1070–8. doi: 10.2460/javma.2004.225.1070, PMID: 15515986

[8] Little D Tomlinson JE Blikslager AT. Post operative neutrophilic inflammation in equine small intestine after manipulation and ischaemia. Equine Vet J. (2005) 37:329–35. doi: 10.2746/0425164054529472, PMID: 16028622

[9] Hopster-Iversen C Hopster K Staszyk C Rohn K Freenman D Rötting AK. Influence of mechanical manipulations on the local inflammatory reaction in the equine colon. Equine Vet J. (2011) 43:1–7. doi: 10.1111/j.2042-3306.2011.00378.x, PMID: 21790748

[10] Kersjes AW Bras GE Németh F van der Velden MA Firth EC. Results of operative treatment of equine colic with special reference to surgery of the ileum. Vet Q. (1988) 10:17–25. doi: 10.1080/01652176.1988.9694141, PMID: 3376407

[11] Hopster-Iversen CCS Hopster K Staszyk C Rohn K Freeman DE Rötting AK. Effects of experimental mechanical manipulations on local inflammation in the jejunum of horses. Am J Vet Res 2014 75(4): 385–391. doi: 10.2460/ajvr.75.4.385, PMID: 24669925

[12] Davis HA Munsterman A. Ileal impaction and jejunal enterotomy in a 4-month-old Arabian filly. Can Vet J. (2012) 53:71–4. 22753967 PMC3239153

[13] Edwards GB. Resection and anastomosis of small intestine: current methods applicable to the horse. Equine Vet J. (1986) 18:322–30. doi: 10.1111/j.2042-3306.1986.tb03642.x 3758013

[14] Parks AH NA ADW Doran RE Baxter GM. Ileal impaction in the horse: 75 cases. Cornell Vet. (1989) 79:83–91. 2912676

[15] Edwards GB. Obstruction of the ileum in the horse: a report of 27 clinical cases. Equine Vet J. (1981) 13:158–66. 7297545 10.1111/j.2042-3306.1981.tb03474.x

[16] Embertson RM Colahan PT Brown MP Peyton LC Schneider RK Granstedt ME. Ileal impaction in the horse. J Am Vet Med Assoc. (1985) 186:570–2. 3988588

[17] Adams SB McIlwraith CW. Abdominal crisis in the horse: a comparison of pre-surgical evaluation with surgical findings and results. Vet Surg. (1978) 7:63–9.

[18] Reeves MJ Curtis CR Salman MD Hilbert BJ. Prognosis in equine colic patients using multivariable analysis. Can J Vet Res. (1989) 53:87. 2914230 PMC1255520

[19] Reeves MJ Curtis CR Salman MD Reif JS Stashak TS. A multivariable prognostic model for equine colic patients. Prev Vet Med. (1990) 9:241–57. doi: 10.1016/0167-5877(90)90070-X

[20] Parry BW Anderson GA Gay CC. Prognosis in equine colic: a study of individual variables used in case assessment. Equine Vet J. (1983) 15:337–44. 6641680 10.1111/j.2042-3306.1983.tb01818.x

[21] Kos VK Kramaric P Brloznik M. Packed cell volume and heart rate to predict medical and surgical cases and their short-term survival in horses with gastrointestinal-induced colic. Can Vet J. (2022) 63:365. 35368402 PMC8922377

[22] Suthers JM Pinchbeck GL Proudman CJ Archer DC. Survival of horses following strangulating large colon volvulus. Equine Vet J. (2013) 45:219–23. doi: 10.1111/j.2042-3306.2012.00620.x, PMID: 22994687

[23] Proudman CJ Smith JE Edwards GB French NP. Long-term survival of equine surgical colic cases. Part 2: modelling postoperative survival. Equine Vet J. (2002) 34:438–43. doi: 10.2746/042516402776117881, PMID: 12358044

